# Sexual health clinic attendees’ views on antibiotic post-exposure prophylaxis and vaccinations for sexually transmitted infections prevention: A qualitative study

**DOI:** 10.1016/j.pmedr.2024.102628

**Published:** 2024-01-26

**Authors:** Alicia J. King, Jade E. Bilardi, Kate Maddaford, Christopher K. Fairley, Eric P.F. Chow, Tiffany R. Phillips

**Affiliations:** a Central Clinical School, Monash University, Melbourne, Victoria, Australia; b Melbourne Sexual Health Centre, Alfred Health, Melbourne, Victoria, Australia; c Department of General Practice, The University of Melbourne, Melbourne, Victoria, Australia; d Centre for Epidemiology and Biostatistics, Melbourne School of Population and Global Health, The University of Melbourne, Victoria, Australia

**Keywords:** Sexually transmitted diseases, bacterial, Sexual behavior, Sexual health, Health knowledge, attitudes, practice, Heterosexuality, Sexual and gender minorities

## Abstract

**Background:**

The rising prevalence of bacterial sexually transmitted infections (STIs) is cause for concern in the context of antimicrobial resistance and the potential health outcomes of untreated infections.

**Objective:**

The Community Awareness and Surveillance of Transmission (CAST) study sought sexual health service users’ views on reducing the prevalence of STIs.

**Methods:**

Semi-structured interviews were conducted with sexual health clinic attendees who had received a diagnosis of chlamydia, gonorrhea or syphilis in the previous six months. Participant comments relating to antibiotic post-exposure prophylaxis (APEP) and vaccination were inductively coded, then compared using comparative qualitative data analysis methods described by Miles and Huberman.

**Findings:**

Twenty-one participants with differing genders, ages, nationalities and sexual orientations, were interviewed. Participants across informant groups expressed concerns about APEP for STI prevention because of potential antimicrobial resistance and personal health impacts. Vaccination against bacterial STIs was more acceptable. Common factors mentioned in relation to both interventions included perception of individual STI risk over time, safety, effectiveness and accessibility.

**Conclusions:**

The views of sexual health service users support efforts to find alternatives to more frequent use of antibiotics, such as vaccinations against bacterial STIs, to reduce STI incidence and support antimicrobial stewardship.

## 1 Introduction

Rising prevalence of bacterial sexually transmitted infections (STIs) demands new approaches to STI prevention. A substantial proportion of these infections occur in the oropharynx presenting challenges due to poor uptake of barrier methods for oral sex and transmission via sexual activities involving saliva (e.g., kissing, spit play) (Chow et al., 2017, Kanmodi et al., 2023, Sarwar et al., 2023). Prophylactic use of doxycycline after potential exposures (i.e., DoxyPEP), has been found to be effective in reducing the likelihood of acquiring an STI in men who have sex with men (MSM) (Luetkemeyer et al., 2023). However, frequent use of antibiotics has been correlated with antimicrobial resistance causing public health concerns, particularly for groups who screen frequently (Kenyon, 2018, Wardley et al., 2023). Vaccinations to reduce the incidence of bacterial STIs are still under investigation but may offer an alternative (Gottlieb et al., 2019).

The Community Awareness and Surveillance of Transmission (CAST) study aimed to explore the perspectives and experiences of a diverse group of sexual health service users on oral STI transmission, testing, treatment and prevention. Findings addressing this broader objective are reported elsewhere (King et al., 2023). A secondary objective was to seek participants’ views on the acceptability of novel interventions for reducing STI prevalence, namely antibiotic post-exposure prophylaxis (APEP) and vaccination, and these views are presented in this paper.

## 2 Methods

A full description of the methods used in this research is available in a previous publication (King et al., 2023).

### 2.1 Recruitment

Participants who met the criteria described in Table 1 were recruited from the Melbourne Sexual Health Centre (MSHC), Australia between February to May 2023. Inclusion criteria were chosen to reflect those at risk of acquiring a bacterial STI and not included in previous research. Eligible MSHC attendees were approached by nurses and, subsequently, contacted via short message service invitation to ensure a diverse sample. Participants provided consent prior to their interview. No participants withdrew after consent. Ethics approval was obtained from Alfred Hospital Ethics Committee (approval no. 43–23).

**Table 1:** Eligibility criteria and sampling framework.

Eligibility Criteria
Attendees of Melbourne Sexual Health Centre
Aged 18 years or older
Tested positive for gonorrhea, chlamydia or syphilis in six months prior
No sex work in the twelve months prior to interview

Sampling framework^a^
Straight (heterosexual) men^b^ and women^c^
Gay, bisexual and other men^b^ who have sex with men
Lesbian, bisexual and other women^c^ who have sex with women
18-24 years old
25-34 years old
Over 35 years old
Australian born
Overseas born

a Groups targeted for minimum numbers of participants.

b Including trans men.

c Including trans women.

### 2.2 Data collection

Once off, semi-structured interviews were conducted by AK, via zoom, telephone or face-to-face in the MSHC clinic, with equal numbers of participants choosing each of these options. An interview guide, described in an earlier publication (King et al., 2023), was used. AK is a cisgender female, PhD-qualified research fellow with experience in studies exploring health service users’ perspectives and experiences. This interest was shared with participants but, otherwise, AK was unknown to participants. Participants were compensated $50AUD (as an e-gift voucher). Interviews were audio-recorded and transcribed, and field notes recorded after each interview. No participants requested a transcript. Five provided feedback on a summary of their interview.

### 2.3 Analysis

Transcripts were coded manually by AK using NVivo 14.2 software. The findings reported in this paper showcase participants’ views relating to the acceptability of APEP and vaccination for STI prevention to sexual health service users. Participant responses to these questions were inductively coded into descriptive codes reflecting their semantic meaning. During coding, three pattern codes (Saldaña, 2016) became evident in the responses of participants representing three broad themes relating to the acceptability of interventions: Reasons supporting use of intervention*;* Conditions limiting the use of the intervention*;* and Concerns preventing their use of intervention.

Participant comments reflecting these degrees of acceptability are summarized in a matrix display (see Table 2) for review and comparison as described by Miles et al. (2020).

**Table 2:** Summary of participant views on antibiotic post-exposure prophylaxis (APEP) and vaccination for bacterial STIs (n = 21).

Prevention measure	Reasons supporting use of intervention	Conditions limiting the use of the intervention	Concerns preventing their use of intervention
APEP	“There's no harm in taking antibiotics” (once a month)* I'm already used to remembering to take tablets (i.e., HIV PrEP^a^)	…I know I've been in a (particularly) high risk situation (e.g., group sex, condom broke, condomless sex)*** …low cost/free** …not too often (i.e., > once a month, > once a year)* …proven effective/preventative* …during a higher risk period of my life* …it doesn't need a prescription/visit to GP^b^/over-the-counter*…part of my regular routine (i.e., APrEP^c^ not APEP) I'd worry about antibiotic resistance if too often (>10 times year)I'd worry about the effects on my gut if too often (>once a month) …not for too long (I'd forget otherwise) … I couldn't access STI testing & treatment (e.g., travelling, living in rural area) …proven safe in clinical trials	I only take antibiotics when diagnosed with bacterial infection)******** I worry about antibiotic resistance****** Antibiotics impact on the good bacteria in your gut** I already take a lot of antibiotics for other health issues** Remembering to take pills is a “hassle”*I'd rather take other preventative measures (e.g., using condoms) * I don't think I'm at enough risk*I don't want to put something in my body unless I need to (e.g., PrEP, anti-depressants) * STIs aren't that serious to warrant prophylactic treatment*I worry about side effects from medications (e.g., PrEP) I get side effects from antibiotics (e.g., gastrointestinal, “thrush”^d^) Antibiotics impact your natural immunity “I don’t know how prophylactic antibiotics work” In my experience, prophylactic antibiotics don’t prevent infections Some antibiotics taste bad You can't drink whilst taking antibioticsI'd have to take them a lot (because of my sexual practices)

Vaccination	I'm pro-vaccination/I'm not an anti-vaxxer***** Prevention is better than cure/I want to protect myself****I've had other vaccines (for STIs^e^)*** I trust there's no harm in it/I trust medical research** I don't have to think about it (i.e., set and forget)**Now I know I'm at risk (having got an STI) * I want to protect othersI know I'm at risk (because of my sexual practices) We could eradicate STIsI got an STI they now vaccinate against (i.e., HPV^f^) I worry about the effects of having repeated STIs It would make me feel more safe continuing to not use condoms I worry about taking antibiotics regularly	…I was engaging in practices I knew were risky*** …proven effective/it reduces my chance of getting an STI*** …I didn't need to get regular boosters*** …it doesn't require multiple doses (i.e., one and done)** …proven safe in clinical trials** …it has minimal side effects** …available* …if low cost/free* …accessible on site (i.e., no prescription required) at sexual health clinic …accessible at my university …it reduces my chance of passing on an STI …my employer requires it …I was in the demographic for it …it covered more than one STI …not a research trial …it doesn't leave a mark on your skin like the [mpox] vaccine. …people I know get it first	I don't think I'm at enough risk** I can take other measures to prevent an STI “Some vaccines can affect your immune system”“I don’t know what's happening inside my body” (when I get vaccine side effects) “I was forced to get the COVID[−19]^g^ vaccine” I got long COVID after the COVID[−19] vaccine I think they rushed the COVID[−19] vaccine

* The number of asterisks represents the number of additional participants sharing the same sentiment.

^ef^ Sexually transmitted infections.

^fg^ Human papilloma virus.

a Human immunodeficiency virus pre-exposure prophylaxis.

b General practitioner.

c Antibiotic pre-exposure prophylaxis.

d Candidiasis.

g 2019 novel coronavirus.

## 3 Findings

### 3.1 Participant characteristics

A diverse group of 21 MSHC attendees participated in interviews of 25–54 min duration (mean = 43, SD = 6.75). Participants’ included straight men (n = 3) and women; (n = 5); gay men (n = 6); bisexual men (n = 2) and women (n = 2); and a man, a woman and a non-binary person who identified with other terms (e.g., queer). They were aged between 22 and 60 years old (mean = 31, SD = 10.85) and seven had arrived in Australia in the past five years.

### 3.2 Participant views

Participant views on the acceptability of APEP and vaccination, as alternative STI reduction measures, are summarized in Table 2. All participant comments on these topics are reflected in one or more codes and individuals’ comments may appear across pattern codes. Commonly expressed views and exemplary quotes are described below. Participant names are pseudonyms.

***Participant views on APEP.*** While there was some variation in participants’ views on APEP, many participants shared reservations and did not know if they would use it. Most commonly, they indicated a preference for taking antibiotics only in the case of a confirmed infection (*n* = 9), citing concerns about antimicrobial resistance (*n* = 7), sometimes in the context of frequent antibiotic treatment for other health conditions (*n* = 3). Forest, expressed a commonly held view:

Knowing about antibiotic resistance and over prescription of antibiotics that is always in my mind. I only ever take them if it’s absolutely necessary….… In this case, I had a confirmed diagnosis so I took them.... Taking antibiotics regularly is an absolutely no-go for me. (Forest, queer man, 25 – 34 years).

The next most common concern related to the impacts of antibiotics on general health, such as gut health (*n* = 4), candidiasis (*n* = 1), effects on general “immunity” (*n* = 1), and a desire to avoid taking medications (*n* = 3). Two participants expressed that the impacts of STIs were not severe enough to warrant prophylactic treatment. Less common were concerns about the inconveniences associated with taking medication (*n* = 3).

***Participant views on vaccination.*** Participants were generally more amenable to vaccination than APEP, with people more likely provide reasons supporting vaccination or describe acceptability based on certain conditions. Participants’ most common reason was being “pro-vaccination” or “not anti-vax” (*n* = 6). In commenting on potential vaccinations for STIs, many participants commented on their experiences with other vaccines: “I took all the COVID [−19] vaccines fine. So I'm not anti-vax or anything. Like I… believe in the science” (Chaton, bisexual woman). Similarly, some received vaccines for other STIs (e.g., mpox) and human papilloma virus (*n* = 4) or reported trusting medical research (*n* = 3). Another commonly expressed view was a preference for prevention over cure (*n* = 5), citing concerns about frequent treatment for STIs or the burden of worrying about one’s STI status: “I was so worried that am I actually getting any of these diseases or not? And if this vaccination can help prevent it, then I would do it” (Zaki, straight woman, 18 – 24 years).

Several participants described the advantage of vaccination over other methods of STI prevention was avoiding the “hassle” of procuring and taking medications, or impacts on the sexual encounter.

It's the same way I have the copper IUD [intrauterine device]. The idea that it can just sit there and I don't have to think about it … I couldn't be bothered to take a pill every day because it meant that I had to remember to do something every day. It's the same reason I probably don't use condoms because I have to remember to either pack one or remember where they are and, in that moment, take the time to step away and to do that. (Rory, queer woman, 18 – 24 years).

***Common factors influencing the acceptability of STI prevention measures.*** Common caveats on acceptability were evident in participants’ views on both APEP and vaccination, within the *conditions limiting the use of the intervention* pattern code (Table 2).

*Perception of individual STI risk.* Perceiving themselves to be at higher risk of an STI, increased the acceptability of STI prevention measures. In the context of concerns about antibiotic resistance and side effects, several participants were willing to consider APEP in instances where they perceived themselves to be at increased risk of acquiring an STI (e.g., group sex, condom broke, condomless sex) or during a higher risk period (e.g., travelling, living overseas).

I’ve had some experiences, you know, a night where I’ve been particularly sexually active with multiple partners. And I’ve decided to take doxycycline 200 mg as a preventative measure… I’ve done it, I think, twice. It would need to be particularly high-risk activity. (Touko, gay man, 35+ years).

The few participants who were willing to take antibiotic prophylaxis on regular basis (*n* = 3) were men (gay and straight) engaged in regular penetrative sex without a condom with casual partners. Two indicated they would be willing to take APEP no more than ten times per year, and one preferred daily pre-exposure prophylaxis, having sustained a period of being STI free as a participant in a DoxyPrEP study: “I went, I reckon, 18 months every single three months I had something, that’s why they called me up for this trial. Yeah, I went for 12 months I did not have an STI” (Nuru, gay man, 35+ years).

Similarly, the acceptability of vaccination was influenced by participants’ use of other prevention measures (e.g., condoms, avoiding casual sex), at a given time.

I’d rather not have the vaccine, and then just back myself to make the right decisions and be sensible enough. But … if I was single and travelling and, you know, in a high risk situation, definitely it would be something that I’d consider more. (Kimball, straight man, 25 – 34 years).

*Effectiveness and safety.* Effectiveness and safety were mentioned in the comments of participants in reference to both APEP and vaccination (see Table 2 – *conditions limiting the use of the intervention* and *concerns preventing their use of intervention*). For antibiotics, concerns about safety related to antimicrobial resistance (*n* = 7), gastrointestinal (n = 3) and other side effects (e.g., candidiasis) (*n* = 2) were more commonly mentioned than concerns about effectiveness (*n* = 2). For vaccinations, participants were generally willing to accept a vaccine that had been found to be safe (*n* = 3) and effective (*n* = 4) in clinical trials but drew on experiences with other vaccines (e.g., mpox, COVID-19) in their desire to avoid side effects.

*Accessibility.* For many participants, their likely uptake of STI prevention measures was also conditional on how easily they could be accessed (see Table 2 – *Conditions limiting the use of the intervention*). Concerns about cost were mentioned in relation to both APEP and vaccination. Related to cost but also convenience, needing to attend a clinic to get a prescription or return on multiple occasions for booster vaccinations was seen as a barrier to access. Conversely, being able to access a vaccine whilst attending for STI screening or on university campus supported their likely uptake.

## 4 Discussion

This study sought sexual health service users’ perspectives on APEP and vaccination for the prevention of bacterial STIs. Findings suggest a preference for vaccinations over APEP, in the context of concerns around antimicrobial resistance, side effects, and the burden of obtaining and taking oral medications.

As reported elsewhere, and by participants in this study, the incompatibility of pleasure with barrier method use for oral sexual practices (Kanmodi et al., 2023, King et al., 2023, Sarwar et al., 2023) underscores the need for prevention measures that do not impact the pleasure of participants during the sexual encounter. The acceptability of STI prevention measures not impacting the sexual encounter (i.e., APEP, vaccination) was described by participants in this research as a function of three factors, namely: their perception of their current STI risk; the perceived safety and efficacy of the intervention; and the accessibility of the intervention.

Both APEP and vaccinations present potential alternatives to frequent testing and treatment for at-risk individuals which has proven largely ineffective to date in reducing community prevalence of STIs (Fairley et al., 2022). Recent research reporting the use of antibiotic prophylaxis, with and without prescription, by 23 % of MSM surveyed (Hornuss et al., 2023) suggests community demand for alternative to barriers methods offering protection against bacterial STIs. However, participants in this study expressed concerns about safety and accessibility of APEP. To the best of our knowledge, this is the first study to compare views on APEP and vaccination. Nonetheless, greater uptake of vaccinations for HPV amongst MSM who had heard about vaccination from more than one source suggest vaccination may be acceptable to those already using APEP, if available (Stearns et al., 2020).

A limitation of recruiting through a single clinic is that we were unable to recruit any women who had sex with women only, trans men or trans women, who are not frequent users of MSHC services. Moreover, this recruitment method excluded those not engaged with sexual health services who may have lower health literacy. Offering participants their preferred choice of interview medium allowed for inclusion of a more diverse sample and resulted in no notable differences in data collection or analysis.

## 5 Conclusions

APEP has been touted by some as a means of reducing STI transmission, in the absence of acceptable and available alternatives. Sexual health service users’ views, however, echo public health researchers’ concerns about the overuse of antibiotics for both treatment and prophylaxis. In contrast, vaccination against bacterial STIs, if proven in clinical trials, was described as a highly acceptable means of STI prevention in terms of perceived safety, and reduced healthcare and emotional burden. These findings urge sexual health researchers and their funders to redouble their efforts towards safer and more acceptable STI prevention measures.

## CRediT authorship contribution statement

**Alicia J. King:** . **Jade E. Bilardi:** Conceptualization, Methodology, Supervision, Writing – review & editing. **Kate Maddaford:** Project administration, Resources, Writing – review & editing. **Christopher K. Fairley:** Conceptualization, Funding acquisition, Supervision, Validation, Writing – review & editing. **Eric P.F. Chow:** Conceptualization, Funding acquisition, Supervision, Validation, Writing – review & editing. **Tiffany R. Phillips:** Conceptualization, Methodology, Supervision, Validation, Writing – review & editing.

## Declaration of competing interest

The authors declare that they have no known competing financial interests or personal relationships that could have appeared to influence the work reported in this paper.

## Data Availability

The data that has been used is confidential.
